# Identification and Development of BRD9 Chemical Probes

**DOI:** 10.3390/ph17030392

**Published:** 2024-03-19

**Authors:** Ester Colarusso, Maria Giovanna Chini, Giuseppe Bifulco, Gianluigi Lauro, Assunta Giordano

**Affiliations:** 1 Department of Pharmacy, University of Salerno, Via Giovanni Paolo II 132, 84084 Fisciano, Salerno, Italy; ecolarusso@unisa.it (E.C.); bifulco@unisa.it (G.B.); glauro@unisa.it (G.L.); 2 Department of Biosciences and Territory, University of Molise, Contrada Fonte Lappone, 86090 Pesche, Isernia, Italy; mariagiovanna.chini@unimol.it; 3 Institute of Biomolecular Chemistry (ICB), Consiglio Nazionale Delle Ricerche (CNR), Via Campi Flegrei 34, 80078 Pozzuoli, Napoli, Italy

**Keywords:** BRD9, inhibitors, small molecules, PROTACs, leukemia, drug discovery

## Abstract

The development of BRD9 inhibitors involves the design and synthesis of molecules that can specifically bind the BRD9 protein, interfering with the function of the chromatin-remodeling complex ncBAF, with the main advantage of modulating gene expression and controlling cellular processes. Here, we summarize the work conducted over the past 10 years to find new BRD9 binders, with an emphasis on their structure–activity relationships, efficacies, and selectivities in preliminary studies. BRD9 is expressed in a variety of cancer forms, hence, its inhibition holds particular significance in cancer research. However, it is crucial to note that the expanding research in the field, particularly in the development of new degraders, may uncover new therapeutic potentials.

## 1. Introduction

Bromodomains (BRDs) represent evolutionarily preserved protein modules with the capability to interpret modifications introduced by histone acetyltransferases (HATs). Specifically, they exhibit selective recognition and binding of ɛ-N-acetyl-lysine residues on histone tails, functioning as readers of the genetic code. Consequently, bromodomains play a crucial role in the regulation of gene expression.

**Figure 1:**
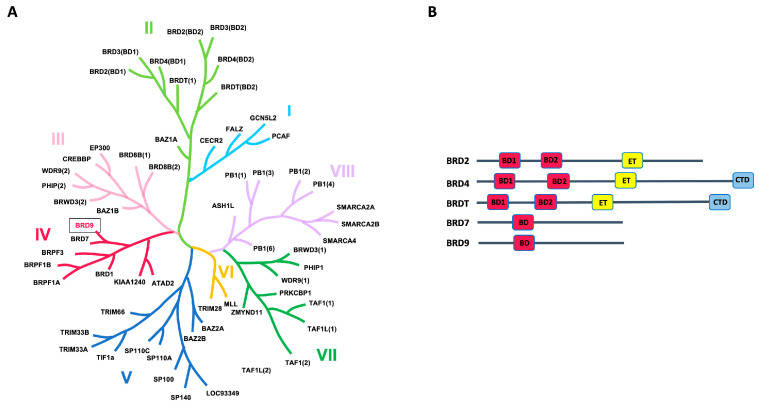
(**A**) Human bromodomain phylogenetic tree. The Roman numerals (I to VIII) represent the different families. (**B**) Modular structure of most representative BET proteins and bromodomain-containing proteins of family IV.

The term “bromodomains” originates from the Brahma gene of Drosophila, in which the bromodomain sequence was initially discovered [1]. The human genome encompasses 61 bromodomains within 46 distinct proteins exhibiting catalytic functions. These proteins are implicated in various pathological processes and are ubiquitously present in most tissues [2]. Seventeen bromodomains are categorized into eight primary families (Figure 1A). Among these, the BET proteins (bromo- and extra-terminal domains) stand out as the most extensively studied. This group includes BRD2, BRD3, BRD4, and BRDT (bromodomain testis-specific protein). These proteins are encoded by paralogous genes, possibly originating from the duplication of an ancestral gene [3].

The BRD7 and BRD9 proteins, which belong to group IV of the family, are strictly related. Both of them are associated with two ATP-dependent chromatin-remodeling complexes, which constitute components of the switch/sucrose nonfermentable (SWI/SNF) complex, crucial for regulating gene expression by modification of the chromatin structure. BRD9 (bromodomain-containing protein 9) is primarily linked to the ncBAF (noncanonical BRG1/BRM-associated factor), consisting of a central ATPase (either BRG/SMARCA4 or BRM/SMARCA2) and several BRG-/BRM-associated factors (BAF subunits), including BRD9 [4]. On the other hand, the PBAF (polybromo-associated BAF) chromatin-remodeling complex, also characterized by its ATPase subunit, incorporates BRD7 instead of BRD9 [4].

These complexes can exhibit distinct or even opposing functional roles. The ncBAF complexes present on chromatin are associated with enhancers, regulating various crucial biological processes, from self-renewal and pluripotency in embryonic stem cells to neural differentiation. Instead, the PBAF complex plays an essential role in maintaining genomic integrity during mitosis [5].

Similarly, BRD9 and BRD7, sharing a certain sequence homology, may have different functionalities or even antagonistic roles. BRD9 demonstrates recognition and binding capabilities for acetylated or butylated lysine residues, leading to post-translational modifications implicated in various diseases, especially cancer. In addition, numerous studies highlight the role of BRD7 as a tumor suppressor gene, implicating it in the p53 and BRCA1 pathways, particularly in the development of breast cancer [4].

BET proteins share a common architecture, characterized by the presence of two bromodomains (BD1 and BD2) and an extra-terminal (ET) domain (Figure 1B). In addition, BRD4 and BRDT contain a C-terminal domain (CTD). Differently, BRD7 and BRD9 proteins contain a unique bromodomain (BD) (Figure 1B). In terms of structural features, all domains share a conserved secondary structure known as a “BRD fold”, characterized by four α-helices (αZ, αA, αB, and αC) and two loops (ZA and BC). The ZA loop connects αZ and αA helices, while the BC loop connects the αB and αC helices [6]. The acetyl-lysine-binding site (Kac region) comprises a conserved asparagine crucial for acetyl-lysine recognition in the ZA and BC loops, a hydrophobic region referred to as the “shelf”, and the initial residue of the alpha helix αC, known as the gatekeeper. In the BET family, the gatekeeper typically consists of an isoleucine-valine, effectively limiting the size of the deep pocket. In contrast, for BRD7 and BRD9, the gatekeeper is a tyrosine residue [6].

Bromodomains are categorized on the basis of the amino acid sequence at the binding site, a crucial factor for ligand interaction. This classification becomes pivotal for evaluating the selectivity of inhibitors [7].

Additionally, each bromodomain has a specific histone peptide as substrate. The investigation into substrate specificity of BRDs has been investigated with different biophysical techniques, such as isothermal titration calorimetry (ITC), fluorescent polarization (FP) spectroscopy, surface plasmon resonance (SPR), and NMR. Subfamily IV of BRDs contains two AAA-domain-containing proteins (ATAD2A and KIAA1240), the bromodomain-containing proteins BRD7 and BRD9, and three bromodomain and PHD finger-containing proteins (BRD1, BRPF1, and BRPF3). The binding of these proteins to specific lysine residues of histone proteins is summarized in Table 1 [7,8,9,10,11,12].

The inhibitors discovered for each bromodomain were evaluated using the same biophysical assays utilized for the specific substrates. Among these, the most commonly employed techniques are certainly AlphaScreen and DSF, which are utilized for screening large libraries of compounds due to their high throughput, high sensitivity, and rapid detection time. In particular, AlphaScreen technology stands out compared to others, as it not only assesses the binding with the protein but also the displacement of the natural ligand from the BRD binding site. However, each biophysical assay is often used in conjunction with orthogonal techniques to avoid false positive results and to validate the binding.

## 2. Bromodomain-Containing Protein 9 (BRD9): An Epigenetic Target Implicated in the Progression of Cancer

**Figure 2:**
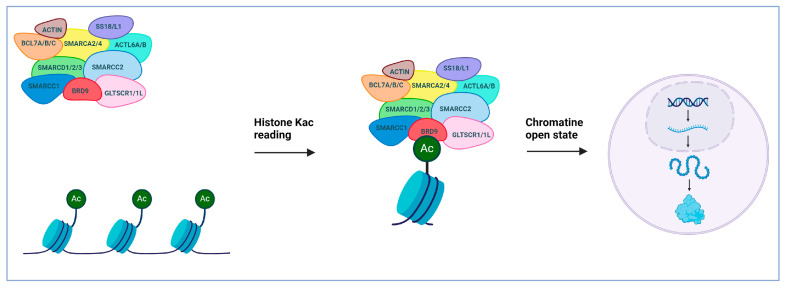
BRD9 in the SWI/SNF remodeling complex orchestrates gene regulation by recognizing acetylated histones, contributing to chromatin accessibility, thus enabling transcription.

This review will specifically focus on bromodomain-containing protein 9 (BRD9). As already mentioned, BRD9 serves as a subunit within the ncBAF complex, and it is classified as a member of bromodomain family IV (Figure 1A) [13]. This protein functions as an epigenetic reader; BRD9 specifically recognizes acetylated lysines of histones and other proteins and recruits the ncBAF complex, thereby controlling gene transcription (Figure 2).

Numerous research studies have consistently disclosed BRD9’s crucial oncogenic role in various cancer types, influencing tumor proliferation and differentiation [4]. Furthermore, BRD9 is intricately involved in multiple signal transduction pathways, chromosomal activities, and nuclear organization [14].

In the Cancer Genome Atlas cohort, a recent study thoroughly examined the genomic changes in the genes encoding histone acetylation modulator proteins (HAMPs). The expression analysis revealed that the majority of HAMPs are widely expressed in all forms of cancer. Among the 63 possible therapeutic target HAMPs found by integrated genome analysis, BRD9 has the highest overall recurrent score, which suggests that this protein is a very attractive target for cancer therapy [15]. Notably, the BRD9-encoding gene, situated on chromosome 5p, exhibits overexpression in cervical cancer [16] and nonsmall cell lung cancer [17]. Moreover, BRD9 has been frequently identified with mutations in squamous cell lung cancer [18], prostatic adenocarcinoma [19], endometrial cancer [20], and hepatocellular carcinoma [21]. Significantly, various pieces of evidence have demonstrated that BRD9 knockdown effectively restrains the proliferation of human acute myeloid leukemia (AML)**,** synovial sarcoma [22], and malignant rhabdoid tumor (MRT) [23]. Despite the acknowledged therapeutic potential of BRD9, the precise biological mechanisms in which it is involved remain elusive.

Therefore, since BRD9 is overexpressed in several types of cancer and involved in the dysregulation of gene expression, inhibiting BRD9 aims to interfere with the processes that promote cancer development and progression. BRD9 inhibitors strive for specificity, meaning they target the BRD9 protein, minimizing the impact on other bromodomain proteins that could have an opposite effect, such as BRD7, which has tumor-suppressive functions. This selectivity can be advantageous in reducing side effects and improving the overall safety profile of the therapeutic intervention. However, it is important to note that, while these potential advantages are promising, the development of BRD9 inhibitors is still an active area of research, and clinical trials are necessary to determine their safety and efficacy in human patients.

## 3. BRD9 Binders

BRD9 has been a target of recent exploration. Only in the last decade has the scientific community uncovered its involvement in cancer diseases and commenced investigations into ways to suppress it. LP99 was the first small molecule used as a BRD9 inhibitor in 2015 [24]. Following this, because of the structural similarity among various bromodomains, numerous nonselective inhibitors were initially developed [25,26,27,28,29,30,31]. I-BRD9, discovered in 2016, represents the first potent and selective inhibitor of this bromodomain, highlighting the distinct activity between BRD9 and BRD7 [32]. All BRD9 inhibitors share the common characteristic of being nontoxic to healthy cells. However, even though many of these inhibitors (both selective and nonselective) prove to be highly potent against the target of interest, sometimes their cellular activity is weak. For this reason, starting from these small molecules, PROTACs (protein-targeted cell activity enhancers) have been developed to enhance cellular activity.

### 3.1. Nonselective Binders

#### 3.1.1. Quinolone Analogues

**Figure 3:**
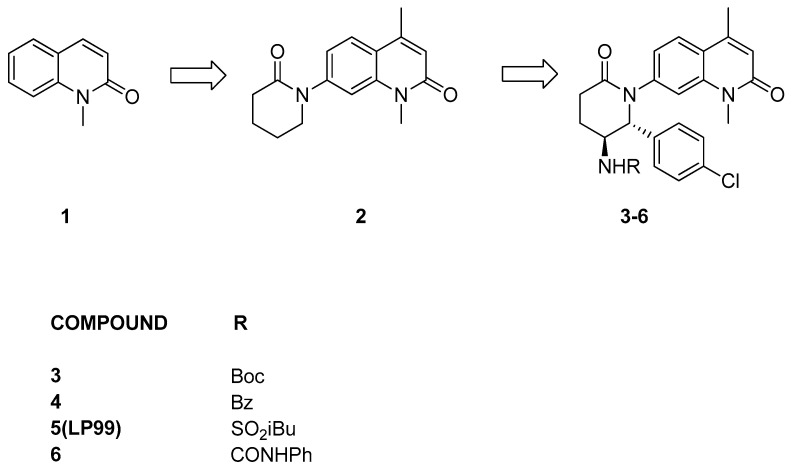
Structure of quinolone binders (compounds **3**–**6**) and their hit compounds (compounds **1** and **2**).

LP99 [24] development originated from a carefully chosen lead compound, 1-methylquinolone (**1**, Figure 3), demonstrated to function as an orthosteric ligand targeting the bromodomain of ATAD2, which shares similarity with the bromodomain of BRD9. This selection was deliberate, as the N-methyl amide portion serves as a mimetic of acetyl-lysine, forming hydrogen bonds with a water molecule and a conserved asparagine. A range of quinolones with diverse N-heterocycles were strategically incorporated at position C7, aiming to capitalize on the hydrophobic cavity between the ZA and BC loops for the selective inhibition of the protein. Among the various evaluated substitutes, valerolactam appears to be the most promising. Then, in order to form interactions with the hydrophobic surface and thereby enhance the compound’s affinity, a methyl group was introduced at position 4 of the 1-methylquinolone (compound **2**). Further modifications were made to the valerolactam group to bolster selectivity: a 4-chlorophenyl group, interacting with another hydrophobic cavity, was introduced at C6, and a carbamate group creating an additional hydrogen bond between the NH of the group and the Gly43 carbonyl group, was added at C5 (compound **3**). Then, the carbamate-protecting group was eliminated, and the resultant amine underwent derivatization through reactions with a variety of acyl chlorides, chloroformates, isocyanates, and sulfonyl chlorides, yielding diverse amides, carbamates, ureas, and sulfonamides for subsequent testing. The three best compounds, **4**–**6**, all with different groups, were generated, and, finally, compounds were resolved into their enantiomers. Notably, compound **5** (Figure 3), also referred to as LP-99, seemed to be the most active of the series, with a K_D_ value of 99 nM against BRD9. Cocrystallization studies revealed that the enantiomer targeting BRD9 is the 2*R*,3*S* configuration [24].

The compound was extensively profiled for BRD selectivity using differential scanning fluorimetry (DSF), revealing remarkable selectivity with minimal stabilization (<1 °C) of all expressible BRDs (48 out of 61 in the human genome), excluding BRD7/9. Finally, cytotoxicity tests in human osteosarcoma U2OS cells for 24 and 72 h indicated the nontoxic nature of the inhibitor at concentrations below 33 μM.

#### 3.1.2. Indolizine Analogues

**Figure 4:**
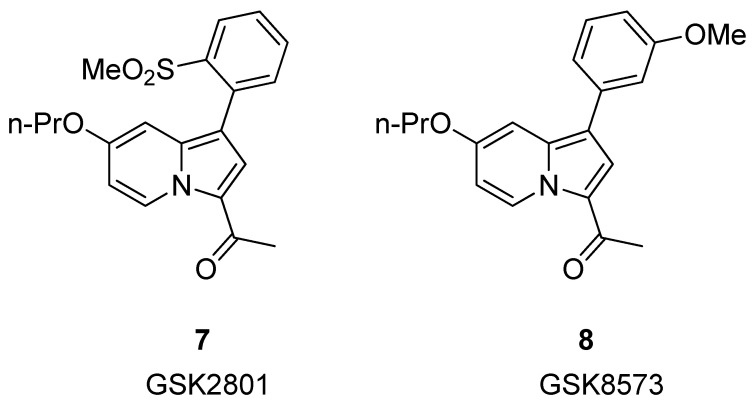
Structure of GSK2801 (compound **7**) and GSK8573 (compound **8**).

In 2015, Chen et al. [33] reported the development of GSK2801 (compound **7**, Figure 4), a potent and cell-active acetyl-lysine competitive inhibitor of BAZ2A and BAZ2B bromodomains. However, during the evaluation of selectivity by ITC, two off-target bromodomains, BRD9 and TAF1(L), were identified (K_D_ = 1.1 and 3.2 μM, respectively). Because of the observed off-target activity with BRD9, they designed a closely related control compound with a highly similar structure (compound **8**, GSK8573), which demonstrated no activity on BAZ2A/B and all other bromodomains, except BRD9 (K_D_ = 1.04 μM).

**Figure 5:**
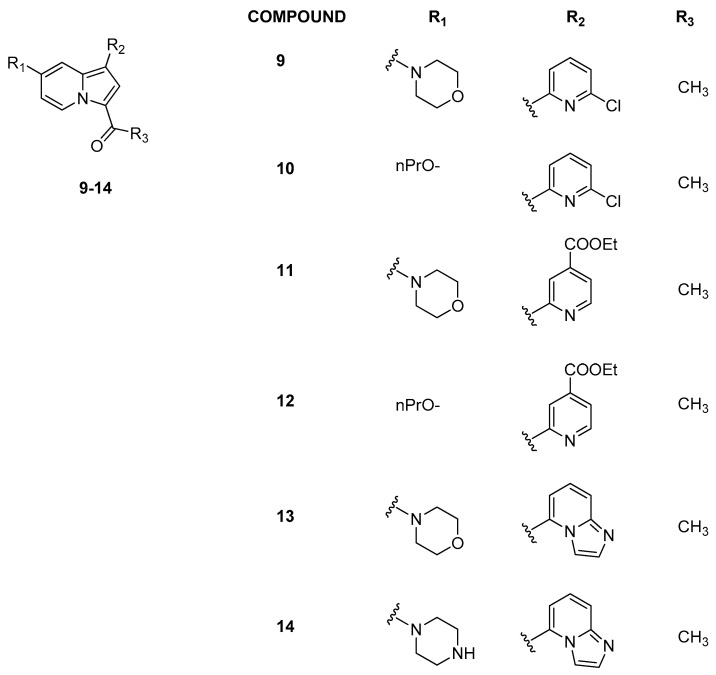
Structure of representative indolizine analogues (compounds **9**–**14**).

Later, Hay et al. [25] moved from the indolizine scaffold of GSK2801 to another series of indolizine derivatives to improve the selectivity toward BRD9 (Figure 5). They found that the introduction of a pyridine ring in position C1 (R_2_, Figure 5) led to an increase in potency and selectivity. Replacing the propoxy ether at position C7 (compounds **10** and **12**) with a morpholine substituent (compounds **9** and **11**) promoted affinity for BRD9, while modifications at R_3_ generally determined a decrease in selectivity. Other analogues were then synthesized, introducing different heterocycles in R_2_ position. Compound **13** with an imidazopyridine in R_2_ position exhibited good affinity for BRD9 (ΔTm = 4.5 ± 0.19 °C), while the C-7 piperazine analogue compound **14** showed poor selectivity against BRD4(BD1) but strong effectiveness against BRD9 (ΔTm = 5.7 ± 0.071 °C). Compound **13** was shown to be very active against BRD9 (K_D_ = 68 ± 4.9 nM) and significantly less potent against BRD7 (K_D_ = 368 nM), with low affinity for BRD proteins of subfamilies I–III and V–VIII. Compound **13** was also evaluated in cellular studies on U2OS cells and, in particular, it exhibited significant inhibitory potency in a ferric-reducing antioxidant power (FRAP) assay [25] involving BRD9/chromatin (Table 2).

#### 3.1.3. From [1,2,4]triazolo[4,3-a]phthalazine Analogues to Bromosporine and Bromotriazine

**Figure 6:**
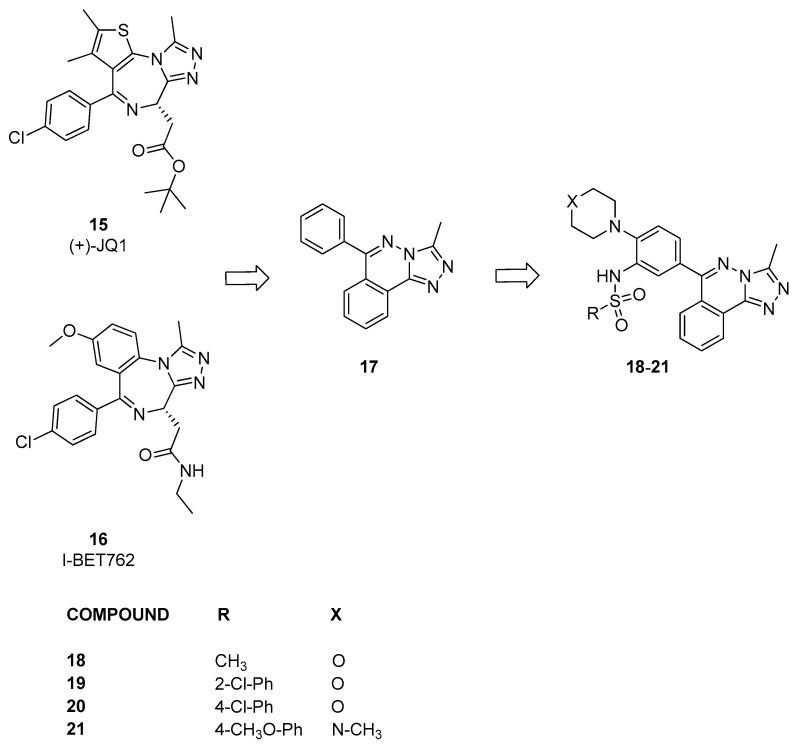
Structure of [1,2,4]triazolo[4,3-*a*]phthalazine analogues (compounds **15**–**21**).

Fedor et al. [26] developed the first example of binders of bromodomains outside the BET subfamily based on the structures and analysis of the binding mode of (+)-JQ1 and I-BET762 (compounds **15** and **16**, Figure 6), which are chemical probes of the BET family. They proposed that by preserving the 3-methyl-[1,2,4]-triazole motif while modifying the fused ring and pendant substituents, it might be possible to discover new compounds that maintained potency toward bromodomains while altering selectivity for non-BET proteins. Initially, several commercially available compounds containing triazole were assessed against 17 bromodomain-containing proteins of the bromodomain family using DSF. From this screening, it emerged that compound **17**, which is only a modest inhibitor, exhibits a certain selectivity for BRD9. On the basis of this result, a series of analogues were generated, including compounds **18**–**21**. When tested using DSF, these compounds also showed some activity against BRD9. Therefore, these data were confirmed through an AlphaScreen assay against four bromodomains. Compound **18** acted as a nonspecific inhibitor, displaying submicromolar IC_50_ values against BRD4, BRD9, CECR2, and CREBBP (pIC_50_ between 6.2 ± 0.62 and 6.8 ± 0.17). In contrast, compounds **19** and **20** exhibited at least 100-fold potency for BRD4, BRD9, and CREBBP compared to CECR2. Instead, compound **21**, showed a slight preference for CECR2 over BRD4 compared to BRD9 and CREBBP.

Moreover, these compounds demonstrated activity in cells, displaying potent cellular inhibition in a CREBBP and chromatin association FRAP model.

A year later, the same group analyzed the common binding modes of histone-derived peptides across different structural classes of BRDs. To create potent nonselective inhibitors, they opted for a similar triazolopyridazine di-cyclic core scaffold [34] based on previously discussed research on the tricyclic chemotype [26].

**Figure 7:**
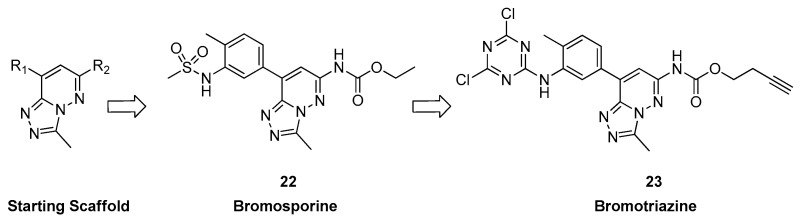
Structure of bromosporine (compound **22**) and bromotriazine (compound **23**).

They reasoned that expanding the scaffold toward the identified binding groove and the BC loop might assist in avoiding subfamily-specific traits. Compounds derived from a specialized library of dicyclic chemotypes, modified at two positions (Figure 7), exhibited widespread activity in a thermal stability assay against diverse human bromodomain targets. Then, through multiple optimization cycles, they identified a potent inhibitor (compound **22**, Figure 7) with nanomolar potency against 13 BRDs and low micromolar activity against 12 additional BRDs. In particular, the best activity was highlighted with CECR2, TAF1(2), BRD4(BD2), BRDT(2), BRD9, and BRD4(BD1) bromodomains (K_D_ = 8.0 ± 1.0, 16.6 ± 2.7, 39.7 ± 2.2, 40.2 ± 2.8, 41.7 ± 3.8, and 41.8 ± 2.8 nM, respectively). They named this versatile inhibitor “bromosporine” (BSP), inspired by the nonselective kinase inhibitor staurosporine [35]. Moreover, they examined the impact of bromosporine on transcription in leukemic cell lines, a cancer type extensively investigated with BET inhibitors [36]. The ultimate findings revealed that BSP demonstrated effects on cell proliferation and clonogenic growth similar to those observed with (+)-JQ1 (Table 3).

After, the goal of D’ascienzo et al. [27] was to develop a new covalent probe for bromodomains by replacing the sulfonamide scaffold of bromosporine with a reactive warhead and introducing a clickable alkyne handle. After discovering a dichlorotriazine reactive moiety capable of selectively labeling lysine residues in HeLa cells, this moiety was selected for the probe [37]. Bromosporine was redesigned to include the chosen warhead and clickable handle. The resulting bromotriazine (compound **23**, Figure 7) was tested for its ability to covalently modify BRDs, and mass spectrometry confirmed the formation of covalent adducts (especially with CECR2, TAF1(1), TAF1(2), BRD7, BRD9, BRD2(BD1), and BRD3(BD1)). Surprisingly, BRD9, lacking a lysine near the KAc binding site, was modified up to 77%. Attempts to map the exact interaction site through LC-MS/MS peptide mapping were inconclusive, prompting a crystallization experiment for further investigation.

#### 3.1.4. Purine Scaffolds

**Figure 8:**
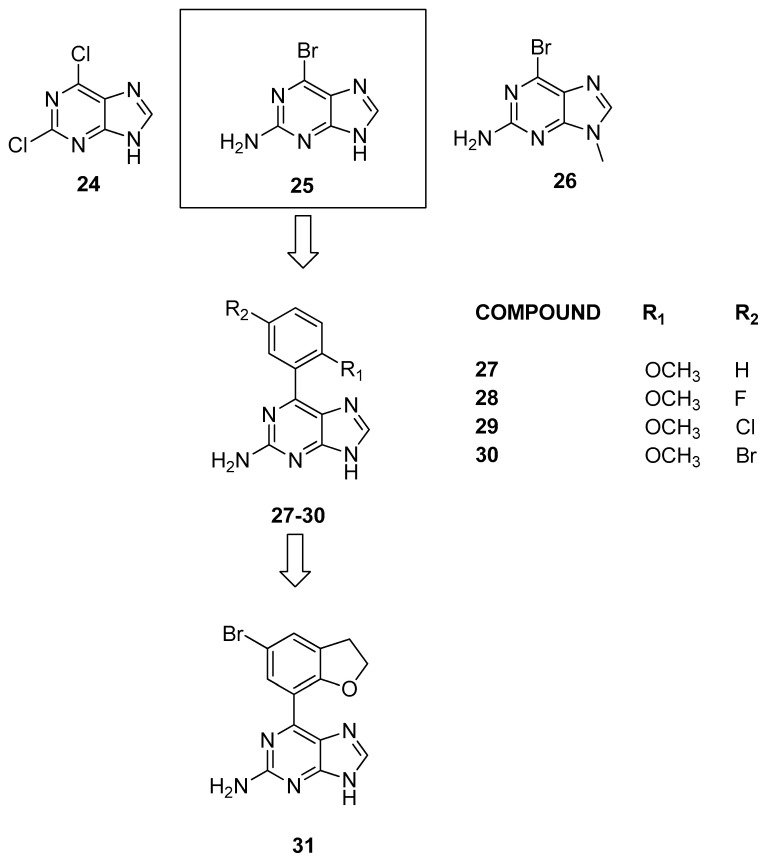
Structure of purine analogues (compounds **24**–**31**).

In 2015, Picaud et al. [28] conducted initial molecular docking experiments on compounds **24**, **25**, and **26** (Figure 8) to assess the interaction of purine fragments with human BRDs. These experiments utilized the crystal structure of the BRD4 complex, and to confirm the computational predictions, a thermal shift assay was used to assess the binding to BRD4 and to five additional BRDs, to cover a broad spectrum of the human BRD phylogenetic tree.

Among these results, the most interesting finding was the interaction between compound **25** and BRD9, especially considering the limited presence of known inhibitors during that period. To delve deeper into the structure–activity relationships of 6-phenyl substituted 9*H*-purines, various compounds with distinct functional patterns were synthesized. Notably, compound **27** demonstrated significant binding to CREBBP when tested by isothermal titration calorimetry (Δt = 1.5 °C) and BRD9 (Δt = 2.9 °C, K_D_ = 641 ± 33 nM).

Subsequently, after a first set of inactive compounds, halide analogues at the meta position, while maintaining the ortho-methoxy functionality (compounds **28**–**30**), revealed improved binding with BRD9 (K_D_ = 351 ± 18, 297 ± 10, and 397 ± 19 nM). However, compounds **29** and **30** were found to be weak binders of BRD4 (K_D_ = 2.04 ± 0.12 and 4.7 ± 0.20 μM). Subsequent substitutions focused on the primary amine, and the addition of a methyl at position 8 did not yield satisfactory results. These results suggest that, in contrast to the previously described ligands, there are two functional groups capable of forming hydrogen bonds with Asn100, as follows: the primary amine and the nitrogen at position 3. Moreover, the functional groups mimicking KAc are unconventional. On the basis of the SAR studies and the evaluation of the crystallographic structure of BRD9, it was hypothesized that increasing the size of the phenyl fragment at position 7 would enhance activity on BRD9. The methoxyphenyl of compound **30** was cyclized to obtain a 2,3-dihydrobenzofuran (compound **31**, Figure 8). Compound **31** confirmed affinity for BRD9 at 278 ± 15 nM, selectively inhibiting chromatin interaction without affecting the histone binding of BRD4, for which compound **30** showed weak affinity (1.37 ± 0.03 µM). Finally, when these compounds were tested in a HEK293 cellular system, they did not exhibit cytotoxicity at concentrations up to 33 μM. Nevertheless, within the same concentration range in bioluminescence proximity assays, they were unable to displace full-length human BRD4 despite the relatively modest in vitro affinity difference between these two proteins.

#### 3.1.5. Alkyl-pyridazin-3(2H)-one Analogs

Clegg et al. [29] started from a previous study [38] on the development of inhibitors targeting the BET family, wherein unpublished data revealed a particular affinity of pyridazinone derivatives for BRD9 and PCAF bromodomains. In light of the high similarity between BRD9 and BRD7, the researchers hypothesized that inhibitors for BRD9 and BRD7 could be developed from the same pyridazinone template.

**Figure 9:**
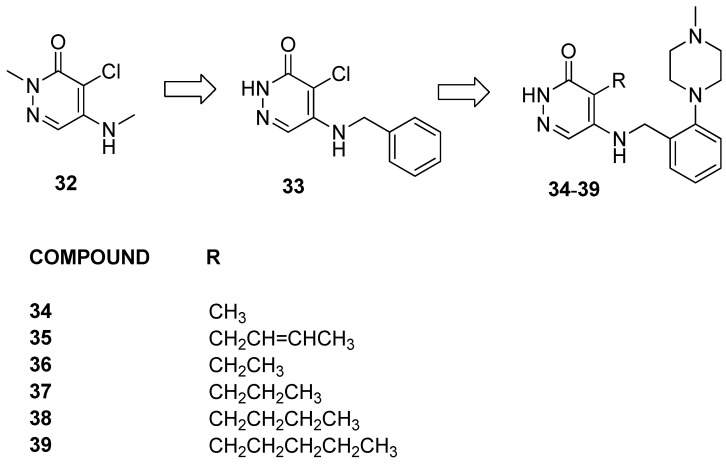
Structure of alkyl-pyridazin-3(2*H*)-one analogues (compounds **32**–**39**).

Compound **32** (Figure 9), reported as being representative of the series, demonstrated activity against both BRD9 and BRD4(BD1) due to a chloro group in the pyridazinone core acting as a KAc methyl mimetic. The substitution of Cl with CH_3_ in compound **33** showed over 100-fold selectivity for BRD9 over BRD4(BD1). Then, to address issues of lipophilicity, the researchers replaced the benzyl ring with polar saturated heterocycles, resulting first in compound **34**. Moreover, the researchers decided to explore the impact of hydrophobic pockets induced by longer lipophilic KAc methyl mimetics.

Compound **35**, bearing an E-crotyl KAc methyl mimetic, showed a ≥40-fold reduction in BRPF1 potency while maintaining BRD9 activity. Exploring other unsaturated groups led to diminished BRD9 potency, underscoring the significance of the KAc methyl mimetic for effectiveness against both BRD9 and BRD4(BD1). Additionally, saturated KAc methyl mimetic derivatives (**36**–**39**) were synthesized and evaluated. Compounds **36** and **37**, bearing an ethyl and a propyl, respectively, were observed to significantly diminish BRPF1 potency while displaying a slight reduction in BRD9 activity. Switching from a three-carbon chain to a four-carbon chain with n-butyl 38 increased BRD9 potency (pKi = 7.2), although the compound remained inactive at BRD4(BD1). Extending the chain with n-pentyl in compound **39** resulted in a reduction in BRD9 potency, accompanied by an unexpected increase in BRD4(BD1) activity. Compound **35** was selected for the evaluation of the selectivity within the broader bromodomain family using the DiscoverX BROMOscan panel. Remarkably, it exhibited high selectivity against the closely related and highly homologous BRD7 (pKi = 6.3), showcasing excellent specificity against the BET family (>×500) and non-BET bromodomains (≥×280). Finally, assessments of cellular permeability demonstrated good permeability with slight intracellular accumulation, underscoring the compound’s potential for effective cellular penetration.

#### 3.1.6. Triazoloquinoxaline Analogues

**Figure 10:**
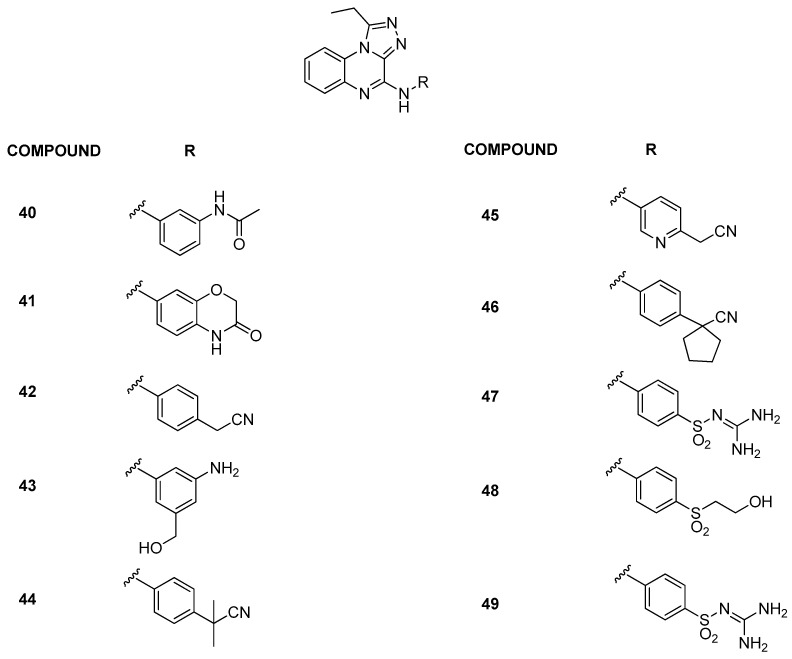
Structures of the first and the second series of triazoloquinoxaline analogues (compounds **40**–**49**).

In 2022, the building of BRD9 structure-based three-dimensional pharmacophore models provided an effective computational implement for the rapid and accurate identification of new potential chemotypes presenting the pharmacophoric features [30]. In particular, the application of the pharmacophore models during an in silico screening applied on an online “Anticancer” and “Anti-Inflammatory” ChemDiv library resulted in the successful identification of compound **40** (IC_50_ = 4.20 ± 1.92 µM) (Figure 10). Starting from the experimental data obtained for compound **40**, the 1-ethyl-[1,2,4]triazolo[4,3-a]quinoxaline moiety (Figure 10) was used for the in silico generation of a combinatorial library of compounds, with the modifications at the C4 position. After in silico investigations and the synthesis of the selected compounds, AlphaScreen technology highlighted the binding with BRD9 for compounds **41***–***43** (Figure 10) (IC_50_ = 10.06 ± 2.60, 4.79 ± 0.49, and 7.48 ± 2.20 μM, respectively). Moreover, in terms of selectivity, compound **42** proved to be the best of the series, as it is not able to bind any of the other investigated bromodomains (BRD2(BD1), BRD2(BD2), BRD3(BD1), BRD3(BD2), BRD4(BD1), BRD4(BD2), BRDT(BD1), BAZ2B, and CREBBP). Compound **43** showed a weak binding with BRD4(BD2) and BRDT(BD1), while compound **40** with BRD2(BD2), BRD3(BD1), BRD4(BD1), and BRD4(BD2). Instead, compound **41** was the less selective of all, being able to bind the bromodomains BRD3(BD1), BRD4(BD1), BRD4(BD2), and BRDT(BD1) with a residual binding percentage of around 50%.

**Figure 11:**
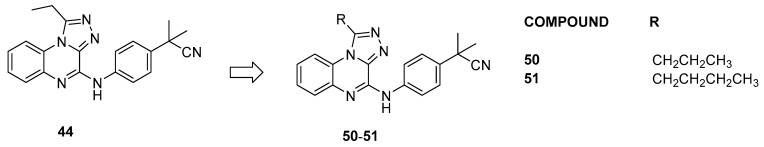
Structures of compounds **50**–**51**.

In a follow-up study [31], a detailed exploration of the triazoloquinoxaline core was conducted by designing and synthesizing new derivatives, maintaining the central core while probing chemical variations around the C-4 position, situated in the ZA loop of the BRD9 binding site. Compounds with amine functionality demonstrated effective binding to BRD9, affirming the importance of the scaffold and NH group (compounds **44***–***49**, Figure 10, IC_50_ between 3.93 ± 0.54 µM and 8.31 ± 0.85 µM). Conversely, the removal or substitution of the linker resulted in a lack of binding. Then, the BromoMELT of compound **44** was performed on all 61 bromodomains as an orthogonal assay to BRD9 and to highlight the binding with other bromodomains. This compound exhibited significant binding affinity exclusively toward BRD9 and two other bromodomains belonging to subfamily 4, BRD7 and BRPF1. In light of this data, to enhance selectivity for BRD9, two new derivatives (compounds **50***–***51**, Figure 11) were designed with propyl and butyl groups at the C-1 position. Despite increased substituent volume, both compounds maintained considerable binding to BRD9. Compound **51**, with an IC_50_ of 6.73 ± 1.55 µM, showed comparable binding to the parent compound **44** (IC_50_ = 3.93 ± 0.54 µM), indicating tolerance for bulkier groups in the hydrophobic anchor region of BRD9.

AlphaScreen experiments confirmed the initial BromoMELT findings, revealing that compounds **44** and **51** bind BRPF1 within the low micromolar range (IC_50_ = 4.50 ± 0.31 µM and 1.48 ± 0.24 µM, respectively). Interestingly, they displayed lower affinity toward BRD7, particularly in the case of **51** (IC_50_ = 18.38 ± 2.06 µM and 95.76 ± 2.74 µM for **44** and **51**, respectively). These findings emphasized the importance of introducing a butyl group at the C-1 position, contributing significantly to the enhanced selectivity toward BRD9 over BRD7.

Then, the bioactivities of compounds **44** and **51** were validated using human leukemia cellular models, including THP-1, Kasumi-1, HL-60, K-562, and CCRF-CEM cells, showing concentration-dependent antiproliferative effects. Moreover, the best IC_50_ value (35 ± 4 µM) was detected for compound **51** on CCRF-CEM cells. These findings suggest prospective anticancer activity warranting further investigation, while no cytotoxicity was observed in healthy human cultures, indicating potential selectivity against high replicative cells.

### 3.2. Selective Binders

#### 3.2.1. Thienopyridone Amidines and Their Amide Analogues

**Figure 12:**
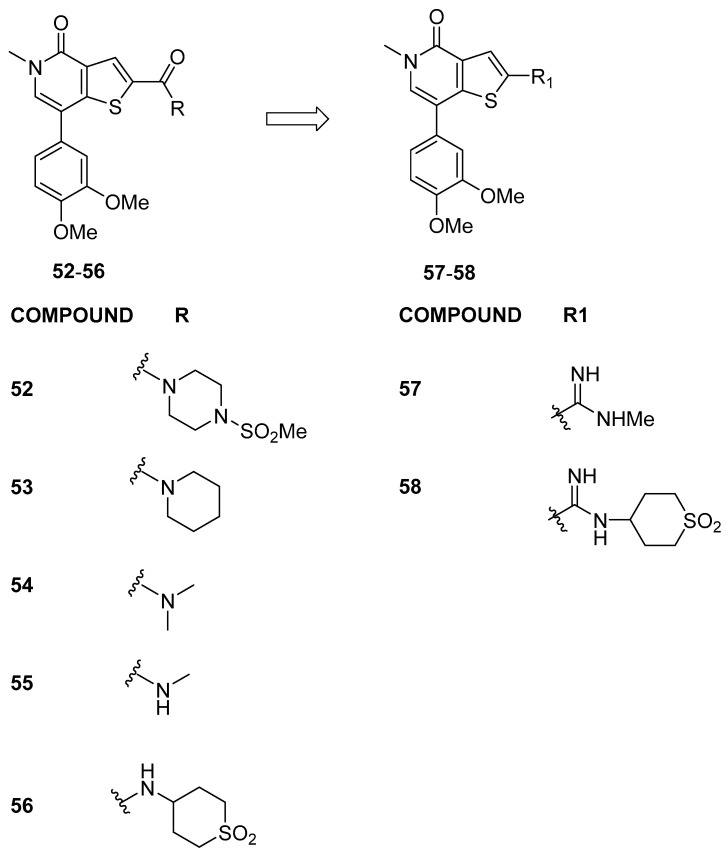
Structures of thienopyridone analogues (compounds **52**–**58**).

Starting from a cross-screening approach involving GSK internal compounds, Theodoulou et al. [32] identified compound **52** (Figure 12), with a thienopyridone scaffold, as a robust binder of BRD9 (as determined by the TR-FRET assay), boasting pIC_50_ values of 6.7 ± 0.12 and 4.7 ± 0.12 against BRD9 and BRD4(BD1), respectively. The potency and selectivity of thienopyridone **52** for the BRD9 bromodomain over BRD4(BD1) were elucidated through X-ray crystal structures for BRD9 and BRD4(BD1), respectively. The thienopyridone ring interactions were found to be similar in both bromodomains. Nevertheless, the detailed interactions of this selective compound exhibit distinctions. Notably, the substitution of BRD9 Ala54/Tyr106 for BRD4 Leu94/Ile46 introduces a differently shaped pocket, causing a tilt of the thienopyridone ring by approximately 10° away from Leu94 in BRD4. This alters the torsion angle of the thiophene 2-position carbonyl bond and influences the position of the pendant piperidinyl sulfonamide. In BRD4, the sulfonamide fails to hydrogen bond to the backbone of Lys141, unlike in BRD9.

To verify whether the amide carbonyl group was responsible for the selectivity over BRD4 of compound **52**, Theodoulou, N.H. et al. conducted investigations into amide modifications. The analysis of the data related to compound **52** revealed the significance of the methyl sulfonamide moiety for BRD9 potency and selectivity over BRD4(BD1). Substituting piperidine in **53** or dimethyl amide in **54** resulted in reduced BRD9 potency, with minimal impact on BRD4 activity. Surprisingly, secondary methyl amide **55**, although not selective over BRD4, restored the BRD9 potency observed in **52**, indicating positive interactions with both bromodomains. Cyclic sulfone amide **56** exhibited a 10-fold improvement in BRD9 activity compared to **55**, although selectivity over BRD4 was limited. X-ray crystallography of **56** in both BRD9 and BRD4 bromodomains provided insights into the observed data, highlighting the importance of amide modifications in influencing interactions and compound efficacy. Unexpectedly, compound **56** exhibited a significantly reduced selectivity window for BRD9 over BRD4 compared to **52**. This is due to the fact that the thiophene and amide of **56** are coplanar in both cases. 

Considering the basic nature of the amidine moiety, the hypothesis was that its charged state would be more favorable in the less hydrophobic environment adjacent to Ala54 of BRD9 than beside Leu94 of BRD4(BD1). Consequently, amidine analogues of secondary amides **55** and **56** were designed and synthesized. Encouragingly, amidines **57** and 58 maintained the BRD9 activity of their direct amide counterparts (compounds **55** and **56**, respectively) while exhibiting improved selectivity over BRD4(BD1). The transformation of methyl amide **55** to amidine **57** resulted in a significant increase in selectivity from 2- to 16-fold. Furthermore, amidine **58** demonstrated a 50-fold selectivity over BRD4, surpassing the 4-fold selectivity observed for its amide analogue **56**.

**Figure 13:**
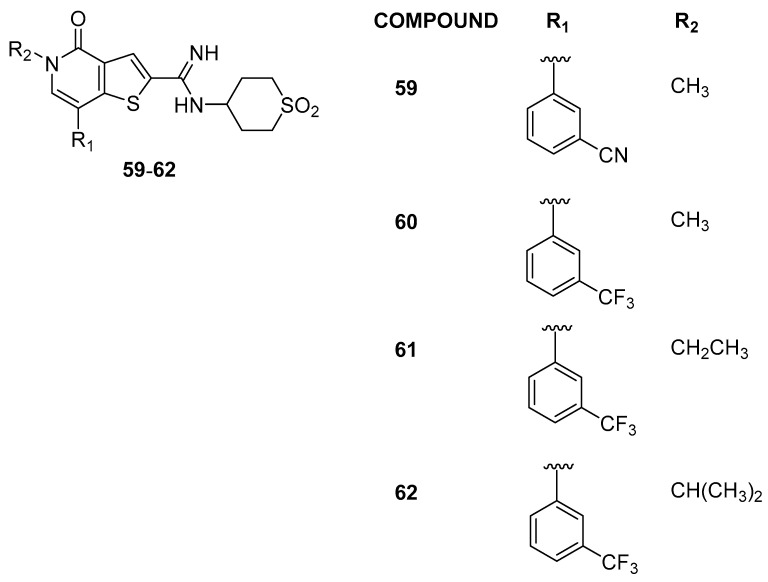
Structures of compounds **59**–**62**.

To further improve the selectivity, modifications were explored at the 7-position of the thienopyridone core, resulting in 7-aryl substituted compounds. Additional modifications with electron-donating and electron-withdrawing substituents on the aryl ring were investigated, with nitrile-substituted compound **59** achieving a 60-fold selectivity, and trifluoromethyl compound **60** attaining a remarkable 160-fold selectivity window (Figure 13). The crystal structures of **60** in BRD9 and BRD4(BD1) explained the observed selectivity, showcasing the significance of electrostatic differences and confirming the role of the amidine. The N-ethyl analogue **61** maintaining ≥100-fold selectivity over BRD4(BD1), while the inclusion of the bulkier isopropyl substituent, compound **62**, led to a decrease in the activities of BRD9 and BRD4. In broader profiling, compound **61** (also known as I-BRD9) demonstrated nanomolar affinity at BRD9 (>700-fold selectivity over the BET family) and 200-fold selectivity over the BRD7 bromodomain; these findings highlight the first selective cellular chemical probe for BRD9. After, Kasumi-1 cells were subjected to treatment with I-BET and I-BRD9. The results indicate that the majority of genes exhibited selective regulation by I-BRD9. Further validation through qPCR identified four genes (CLEC1, DUSP6, FES, and SAMSN1) significantly downregulated by I-BRD9, without a similar effect by I-BET [32].

#### 3.2.2. Isoquinolinone or Pyridinone Analogues

**Figure 14:**
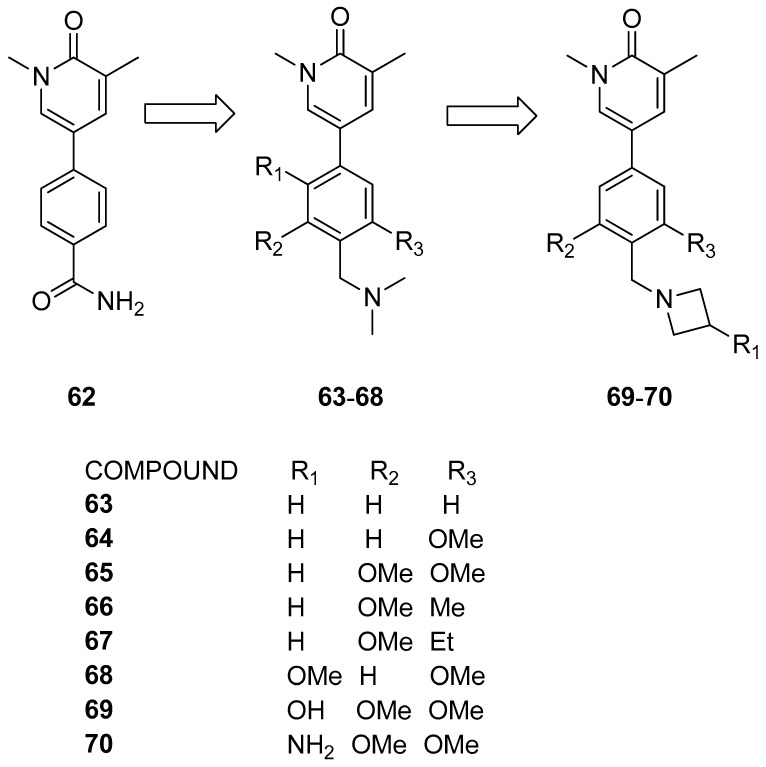
Structures of the first series of pyridinone analogues (compounds **63**–**70**).

Thanks to a combined screening involving DSF, surface plasmon resonance (SPR), microscale thermophoresis (MST), and heteronuclear single-quantum coherence nuclear magnetic resonance (^15^N HSQC NMR) assays, Martin et al. [39] identified the methylpyridopyrimidinone and dimethylpyridinone scaffolds as promising starting points for the development of new BRD9 inhibitors. The chosen primary compound **62** (Figure 14) engaged in T-stacking with Phe44, leading to the substitution of the amide group with methylene dimethylamine (compound **63**), resulting in an eight-fold increase in potency. The incorporation of additional electron-donating groups on the phenyl ring (compounds **64**–**68**) further heightened potency by strengthening T-stacking with Phe44. These modifications aimed to optimize the edge-to-face interaction for enhanced compound efficacy. Examining alterations in the ZA linker region, researchers noted enhanced selectivity toward BRD9 in comparison to its BRD7 counterpart. Potency enhancement was achieved by addressing the backbone carbonyl of His42 with hydroxyl or amine moieties (compounds **69** and **70**). The azetidine substituent proved optimal for interacting with the His42 carbonyl without disrupting T-stacking with Phe44. Compound **70** exhibited low nanomolar activity (IC_50_ = 9 nM) and displayed an induced fit with Phe47, contributing to improved selectivity against BRD4(BD1).

**Figure 15:**
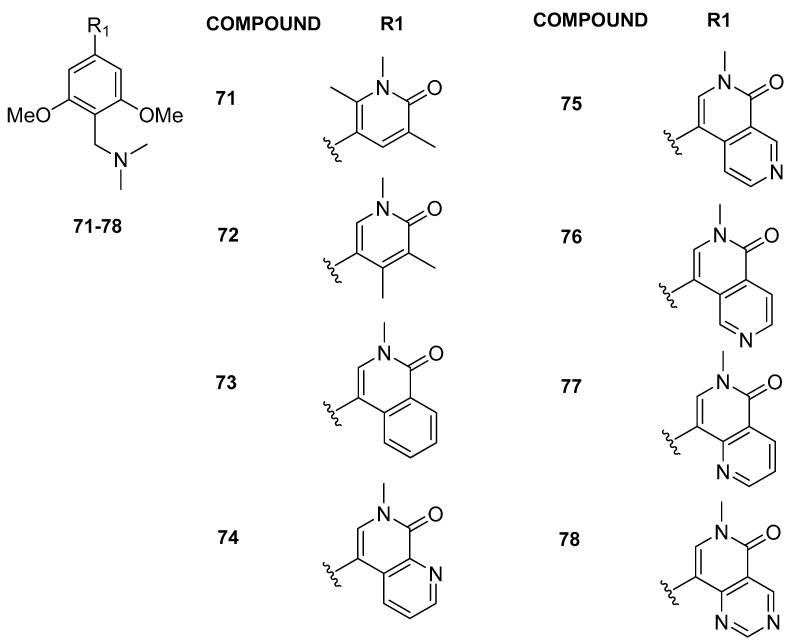
Structures of the second series of pyridinone (compounds **71** and **72**) and isoquinoline analogues (compounds **73**–**78**).

Further modifications in the anchor region, introducing substituents at the 4 or 6 position on the pyridine-2-one core, led to enhanced selectivity against the BET family (compounds **71** and **72**, Figure 15). Employing a comparable strategy, the introduction of an aromatic ring into the pyridinone scaffold enhanced selectivity against the BET family, augmenting the π-stacking interaction with Tyr106 in the BRD9 anchor region for compounds **73**–**78**. The most effective inhibitor, 2-methyl-2,7-naphthyridin-1-one compound **75** (BI-7273), demonstrated a 3-fold increase in affinity for BRD9 and an impressive 50-fold increase in selectivity against BRD4(BD1) compared to compound **65**. Compound **75** additionally established a positive interaction with the carbonyl of Asn100 in BRD9, facilitated by the nitrogen atom at position 7 on the naphthyridinone ring. This interaction involved the acidification of the CH bond at position 8, enabling engagement with the carbonyl side chain of Asn100. Finally, compound **79** was obtained, which exhibited slightly lower potency against BRD9 compared to **75**, but demonstrated enhanced selectivity, being 45-fold more potent for BRD9 compared to BRD7.

**Figure 16:**
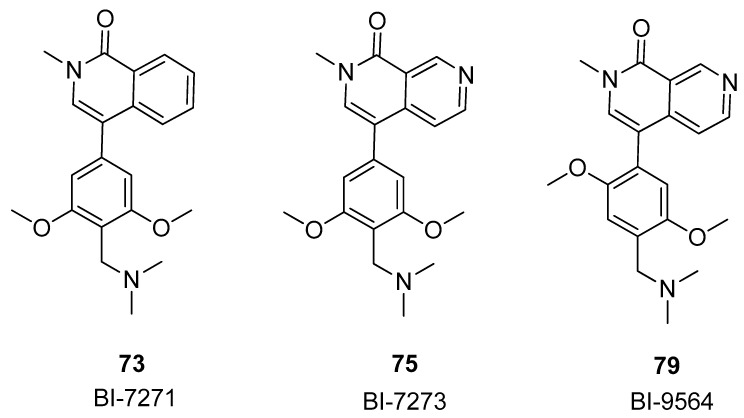
Structures of the most active compounds among the isoquinolinone and pyridinone analogues: BI-7271 (compound **73**), BI-7273 (compound **75**), and BI-9564 (compound **79**).

In conclusion, the compounds **73**, **75**, and **79**, also named BI-7271, BI-7273, and BI-9564 (Figure 16), were revealed through the expansion of the dimethylpyridinone structure to design ring-fused compounds. These compounds exhibited robust potency and selectivity for BRD9 and displayed antitumor activity in an AML xenograft model.

#### 3.2.3. Imidazo[1,5-a]pyrazin-8(7H)-one Derivatives

**Figure 17:**
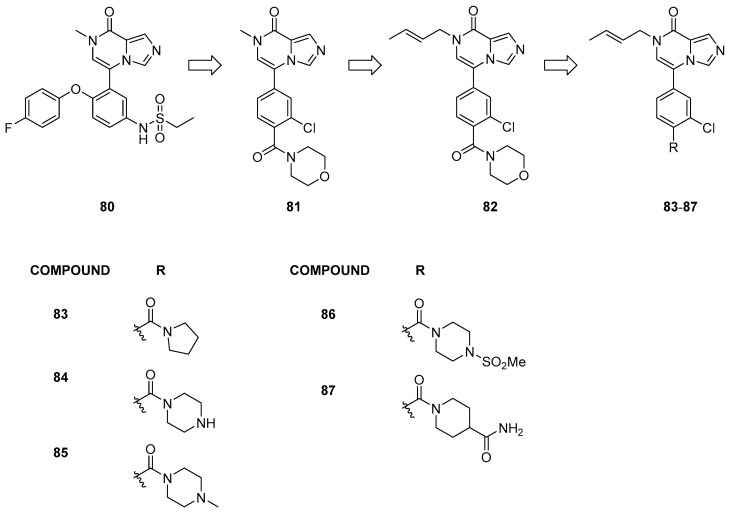
Structures of imidazo[1,5-a]pyrazin-8(7*H*)-one derivatives (compounds **80**–**87**).

Starting from the structure of compound **80** (Figure 17), initially developed as a BRD4 inhibitor (IC_50_ = 30 nM) [40], Zheng et al. [41] conducted structural modifications to enhance activity against BRD9 (IC_50_ = 10 µM). The authors designed a new series of derivatives based on the imidazo[1,5-a]pyrazin-8(7H)-one chemical scaffold by substituting the heterocyclic bicyclic ring with various groups previously reported for BRD9 inhibitors.

These compounds exhibited moderate inhibitory activity against the target protein (IC_50_ ranging from 1.502 ± 0.421 to 7.410 ± 0.913 µM). Compound **81**, the most promising of the series, served as the lead compound, and different alkyl groups on the nitrogen of the imidazo[1,5-a]pyrazin-8(7H)-one moiety were assessed. Among these, compound **82** demonstrated the highest BRD9 inhibitory activity, achieving a 91% inhibition rate at 1 μM and an IC_50_ of 465 ± 13 nM. Its activity is attributed to the presence of double bonds on the alkyl group on the nitrogen and chlorine instead of fluorine in the meta position of the aromatic ring compared to the other compounds. Additionally, it was determined that chlorination in the meta potion was preferable to that in ortho.

Furthermore, modifications to the para position on the benzene ring led to a noticeable increase in the IC_50_ values (compounds **83**–**87**). Compounds featuring piperazine or its derivatives as substitutions exhibited enhanced activity. Notably, compounds **84** and **85** demonstrated significant potency in inhibiting BRD9, with IC_50_ values of 35 ± 7 and 103 ± 16 nM, respectively. All these exemplary compounds exhibited solely BRD9 inhibitory activity without demonstrating any BRD4 inhibitory effects.

Moreover, compound **84** displayed potent inhibition of cell proliferation in the A549 and EOL-1 cell lines (IC_50_ = 6.12 ± 0.18 μM and 1.76 ± 0.05 μM, respectively).

#### 3.2.4. Pyrrole Analogs

A set of 4-acyl pyrroles [42] was initially designed for BRD4(BD1), and specific modifications were introduced to create active small molecules without solubility issues. Binding assays among bromodomain proteins revealed their affinity for the BET family proteins and BRD7, BRD9, BRPF1B, and CBP, as additional targets [42].

**Figure 18:**
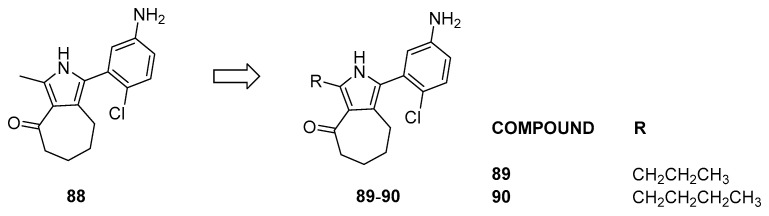
Structures of pyrrole analogs (compounds **88**–**90**).

Notably, compounds containing a sulfonamide moiety display K_D_ values on BRD9 ranging between 67 and 530 nM. Compound **88** emerged as the most effective when evaluated on BRD9, prompting the design of close derivatives (compounds **89** and **90**, Figure 18) to enhance specificity toward BRD9 [43].

Compounds **89** and **90**, compared to the lead compound **88**, showed a 150-fold increased selectivity toward BRD9 compared to BRD7 (for compound **88**, BRD9 K_D_ = 150 nM, BRD7 = 1100 nM, BRD4 = 6900 nM, and BRPF1b = 7000 nM; for compound **90**, BRD9 Kd = 800 nM, BRD7 > 1100 nM, BRD4 > 6900 nM, and BRPF1b > 7000 nM). The binder has a broad spectrum of activity, showing inhibition of growth (GI_50_) in nanomolar values in different cancer cell lines, such as SNB-75, UO-31, CAKI-1, DU-145, SW-620, and NCI-H460.

#### 3.2.5. 6-Methylquinazolin-4(3H)-one Analogs

**Figure 19:**
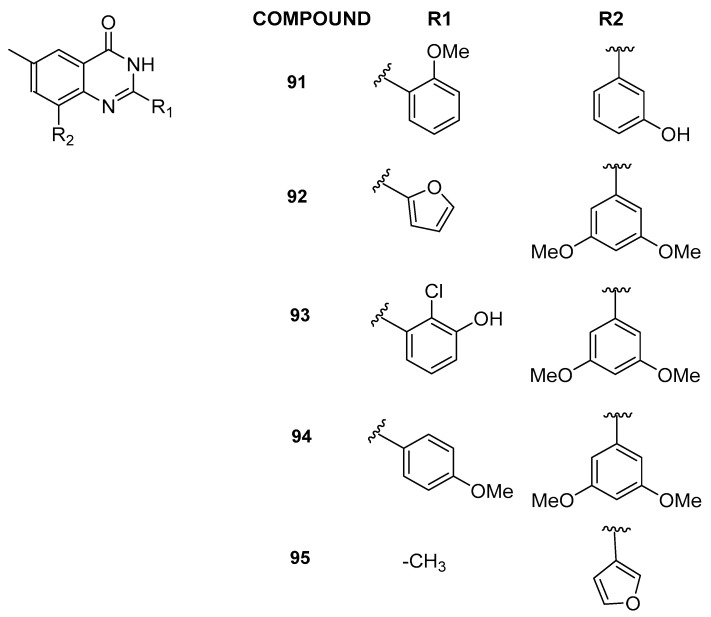
Structures of 6-methylquinazolin-4(3*H*)-one analogues (compounds **91**–**95**).

In 2022, through a multidisciplinary scientific approach integrating computational techniques, synthesis, and biological evaluation, new and selective inhibitors targeting BRD9, characterized by a 6-methylquinazolin-4(3*H*)-one chemical core, were discovered [44]. Specifically, utilizing a study by Combiglide [45], the 6-methylquinazolin-4(3*H*)-one was in silico functionalized with approximately 3000 commercially available benzaldehydes at position 2 and 570 boronic acids at position 8. This process yielded a large virtual library, which was further refined through in silico processing before undergoing molecular docking experiments. Drawing upon the recently developed 3D structure-based pharmacophore models specific to BRD9 [30], 16 compounds were identified for the synthesis step. Notably, compounds **91**–**95** (Figure 19) emerged as particularly potent binders to BRD9, exhibiting efficacy at low micromolar concentrations (IC_50_ between 2.5 ± 0.4 and 10.9 ± 0.3 µM). Additionally, given that quinazolin-4(3*H*)-one derivatives had already been documented as binders for the BET family member BRD4 [46], compounds **91**–**95** were assessed for their interaction with BRD4(BD1). Intriguingly, these compounds did not demonstrate significant binding to BRD4(BD1), establishing them as novel BRD9 binders with promising selective behavior.

#### 3.2.6. 2,4,5-Trisubstituted-2,4-dihydro-3H-1,2,4-triazol-3-one Analogs

**Figure 20:**
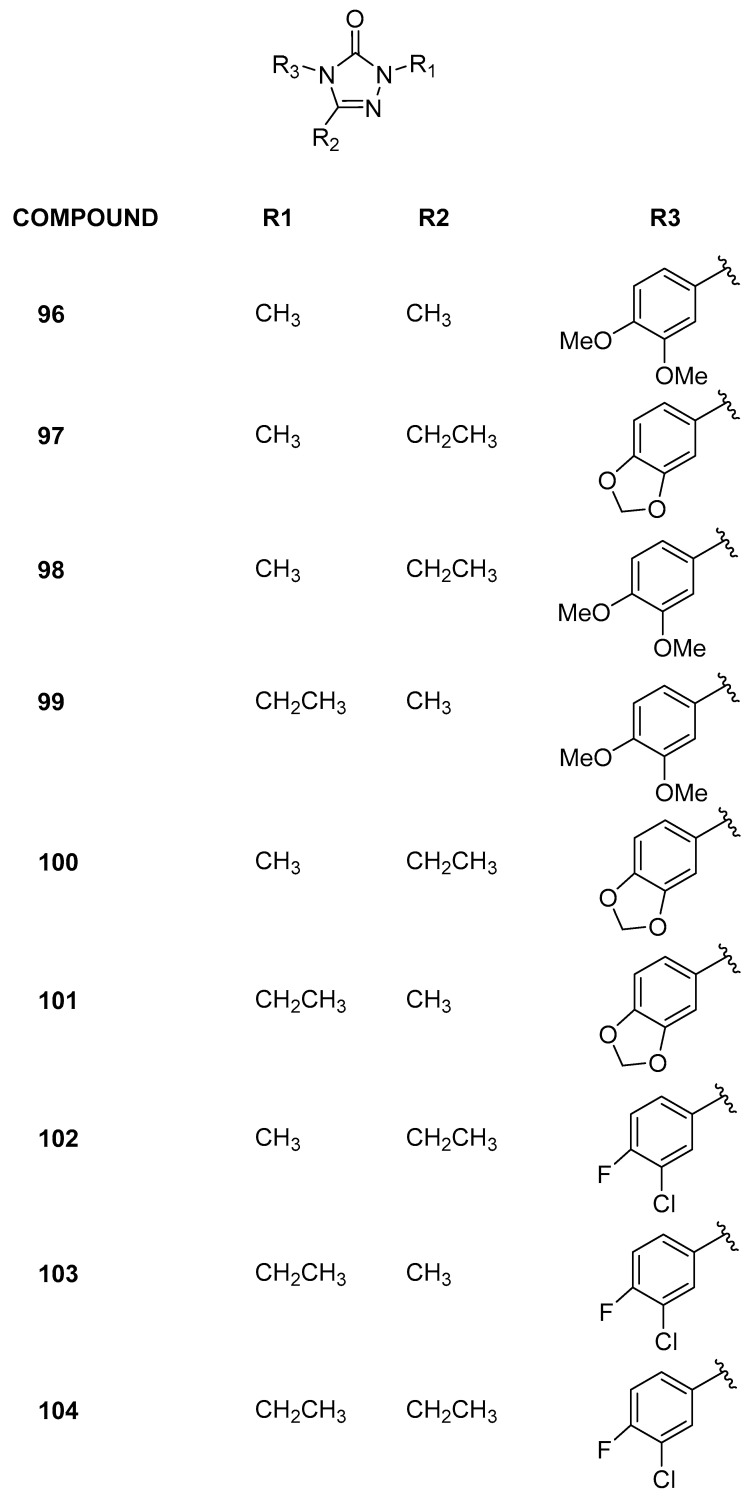
Structures of the 2,4,5-trisubstituted-2,4-dihydro-3*H*-1,2,4-triazol-3-one analogues (compounds **96**–**104**).

Recently [47], employing computational methodologies, selective BRD9 inhibitors with a 2,4,5-trisubstituted-2,4-dihydro-3*H*-1,2,4-triazol-3-one chemical core were identified (Figure 20). Initially, six differently functionalized cores were virtually examined to assess their potential to bind the target. Subsequently, these six scaffolds were combined with 316 aryl isocyanates commercially available to introduce chemical variability at position 4, using the same computational workflow as previously discussed. Among the twenty-one molecules selected and synthesized, two (**96** and **97**) exhibited IC_50_ values in the low micromolar range (0.14 ± 0.03 and 0.35 ± 0.18 µM, respectively). To rationalize the biophysical data and design additional derivatives, the 3D structure-based pharmacophore model was applied, and seven of these new derivatives (**98**–**104**) effectively bound BRD9. Encouraged by these results, the selectivity of all the active compounds was evaluated across a panel of nine BRDs covering most of the related human phylogenetic tree (BRD2(BD1), BRD2(BD2), BRD3(BD1), BRD3(BD2), BRD4(BD1), BRD4(BD2), BRDT(BD1), BAZ2B, and CREBBP). In addition, for compound **97**, the selectivity was further assessed on eight additional bromodomains (including ATAD2, CECR2, SMARCA4, TAF1(BD2), BRPF3, BRD1, BPTF(BRD), and BRD7). This compound exhibited no binding with the most considered bromodomains (residual protein activity percentage between 92.6 ± 3.1 and 100%). However, with BRD7 and TAF(1), this percentage dropped to 75.8 and 77.7, respectively. The IC_50_ evaluation revealed that compound **97** is approximately 60 times more potent on BRD9 (0.14 ± 0.03 µM) compared to BRD7 (71.4 ± 1.1 μM) and similar to TAF(1). Moreover, all the active compounds were evaluated on a selected panel of human cells, composed of both healthy (keratinocytes and enterocytes) and cancer (leukemia, breast, melanoma, and colorectal) cell lines. At the highest concentrations tested, no cytotoxic responses were observed in healthy cells, with IC_50_ values exceeding 500 µM, indicating an absence of detectable biological effects. Preliminary data on human cancer models revealed a notable selectivity in action on leukemic cells. Notably, compounds **96** and **97** demonstrated IC_50_ values below 150 µM on Jurkat cells, indicating a modest yet noteworthy antiproliferative activity that warrants further investigation.

### 3.3. Summary of Cellular Assays

In the first works, the cellular assays were performed through the fluorescence recoveries after photobleaching technique (FRAP) by disruption of the interaction of full-length green fluorescent protein or full-length GFP-tagged BRD9 with acetylated chromatin. With this technique, the displacement of proteins from chromatin was observed, as evidenced by rapid recovery after bleaching. In the case of BRD9, cells were treated with the histone deacetylase inhibitor SAHA (suberoylanilide hydroxamic acid) to elevate acetylation levels and enhance binding. When examining a representative selection of bromodomains in the presence of inhibitors, a significant reduction in fluorescence recovery times was noted, indicative of the inhibition of chromatin-BRD interaction (Table 2).

For compounds **5** and **31** the cytotoxicity assay was also reported. Compounds were tested in different cell lines but in both cases they do not appear cytotoxic at a concentration lower or equal to 33 µM (Table 2).

In the most recent studies, the antiproliferative biological effects were assessed. This involved determining the IC_50_ (the concentration of a drug or inhibitor needed to inhibit a biological process or response by 50%) or EC_50_ (the concentration effective in producing 50% of the maximal response and is a convenient way of comparing drug potencies), or GI_50_ (the dose that inhibits the growth of cells by 50%) values (Table 3) on different cancer cell lines, in which BRD9 is more prominently expressed.

Nevertheless, numerous inhibitors have only been characterized through binding assays on BRD9 or assessments of selectivity against other bromodomains; cellular experiments have not consistently been conducted. Moreover, even though BRD9 operates within a complex, compounds exhibiting significant activity on isolated BRD9 protein might not translate into favorable outcomes in cell assays. To boost cellular activity and thus enhance biological impact, the most promising binders are presently serving as a starting point for chemical degraders (e.g., PROTACs) development.

## 4. BRD9 PROteolysis TArgeting Chimeras (PROTACs)

The conventional drug discovery paradigm has long relied on small molecules binding to specific sites on target proteins to modulate their activity [48]. However, many proteins lack such binding sites or catalytic activity, rendering modulation challenging [49]. Additionally, some proteins operate within large complexes that can compensate for reduced activity due to inhibition. Notably, PROteolysis TArgeting Chimeras (PROTACs) represents a revolutionary approach, particularly in cancer treatment, within the realm of small molecule therapeutics. PROTAC technology offers a novel strategy for drug discovery, capable of targeting proteins previously deemed undruggable by conventional methods [50,51,52]. More specifically, PROTACs are bifunctional degraders that hijack the intracellular ubiquitin-proteasome system (UPS) to induce the degradation and elimination of a target protein. An E3 ubiquitin ligase ligand is linked to a protein-of-interest ligand (also known as a “warhead”) by a linker of varying lengths and chemical composition [53]. BRD9 inhibitors often demonstrate poor cellular activity [47] despite strong activity on the protein, attributable to BRD9’s involvement in the SWI/SNF complex. Consequently, developing PROTACs from these known inhibitors may prove crucial in enhancing their biological efficacy on the target of interest, offering promising avenues for therapeutic intervention.

**Figure 21:**
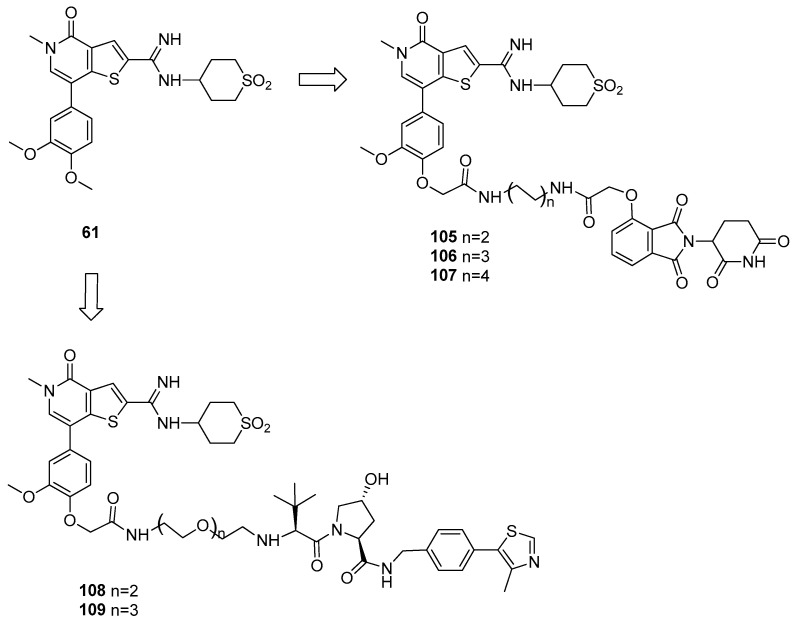
Structures of compounds **105**–**109**.

The first BRD9 heterobifunctional degrader [54] was developed using as a starting point the thienopyridone analogue of I-BRD9 (compound **61**, Figure 21), which displayed high binding affinity (IC_50_ = 7.9 nM) and the possibility of anchoring the E3 ligand by an ether linker attached to the methoxy substituent [28]. A first series of ligands was synthesized, exploring both binding to CRBN and VHL E3 ligase. Products were tested in human AML cell line for 4 h, and BRD9 protein levels were detected by immunoblot. Among the CBRN ligand-based compounds, the one presenting a linker with intermediate length resulted as the most promising (**106**), leading to the most efficient protein degradation; on the contrary, VHL-ligand conjugates (**108** and **109**) failed to induce BRD9 degradation.

**Figure 22:**
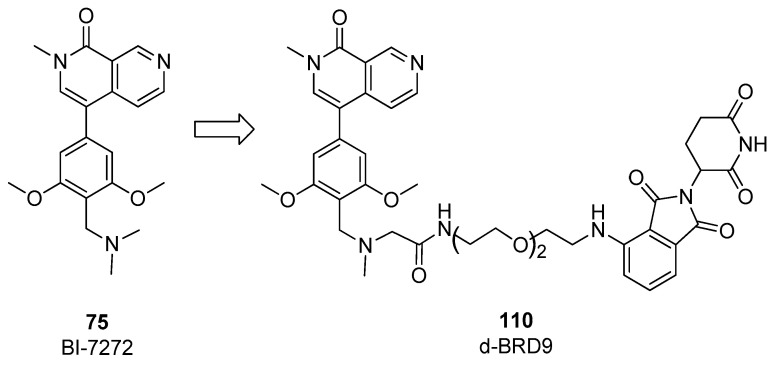
Structure of compound **110**.

To further expand the investigation, in the same work [54], other degraders based on the structure of the binder BI-7273 (compound **75**) and pomalidomide as CBRN ligand were prepared. The impact of nature and length of the linker was also evaluated to improve biochemical and cellular selectivity. Compound **110** (Figure 22) with PEG-linked pomalidomide conjugate (dBRD9) was able to efficiently degrade BRD9 in the MOLM-13 cell line, with reduced off-target binding activity toward other bromodomain containing proteins. The antiproliferative activity of dBRD9 was tested in human AML lines (EOL-1, MOLM-13, MV4), showing large improvement with respect to classical binders I-BRD9 and BI-7273.

**Figure 23:**
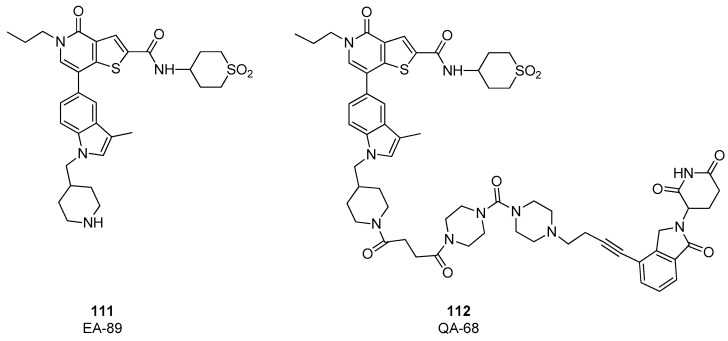
Structures of EA-89 (compound **111**) and QA-68 (compound **112**).

In 2022, on the basis of the thienopyridone scaffold of I-BRD9 (compound 61), a novel binder, named EA-89, was developed, (compound **111**, Figure 23), [55] in which the propyl group linked to pyridone nitrogen accommodates in a hydrophobic pocket acetyl lysine pocket, and the 3-trifluoromethylphenyl ring is substituted with a 3-methylindolic group, that serves as the site for anchoring the linker. EA-89 was then used as a warhead to obtain a cereblon-targeting degrader QA-68 (compound **112**, Figure 23). Both the binder and the PROTAC were tested on several cellular lines of hematological malignancies, showing a strong selectivity for AML as well as B cell acute lymphoblastic leukemia (B-ALL) and multiple myeloma (MM). Interestingly, no impact on the viability of cell lines of the T lymphocytic lineage (acute monocytic leukemia and erythroleukemia) was observed, revealing a BRD9 dependency in acute leukemia and MM [55].

**Figure 24:**
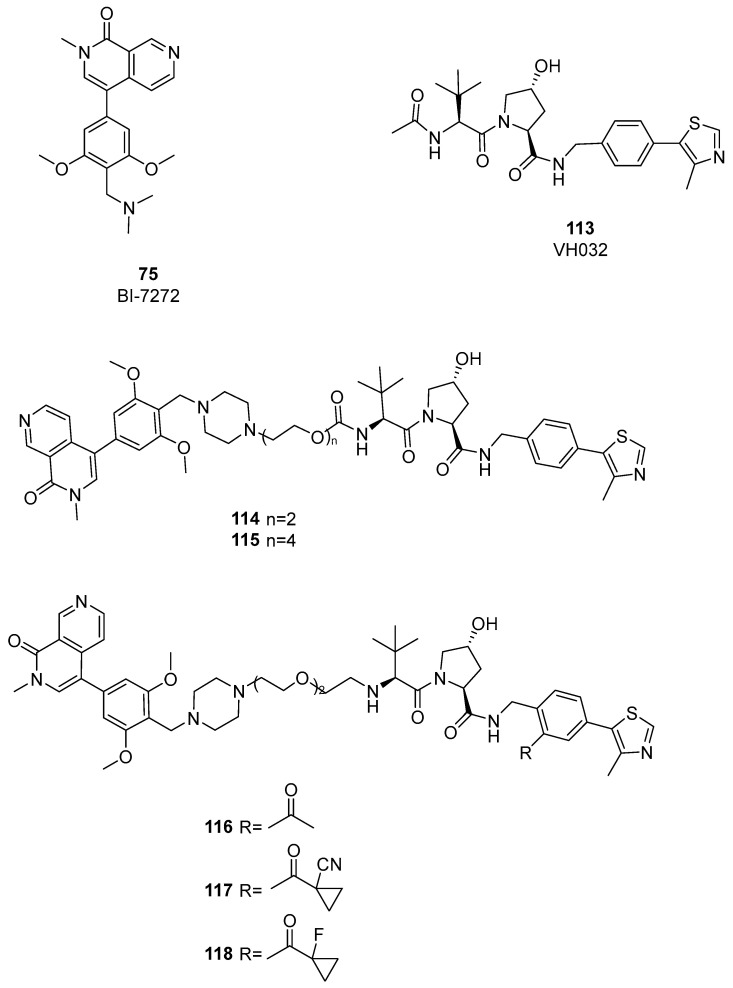
Structures of compounds **113**–**118**.

As highlighted in the study by Remillard et al. [54], CRBN-based PROTACs can exhibit off-target degradation of other proteins. From this perspective, Ciulli’s group developed the first VHL ligand-based dual BRD7/BRD9 degrader [56]. A first set of compounds was synthesized using PEG linkers to connect a BRD binder in which a piperazine group replaced the dimethylamine group of compound BI-7273. As E3 ligase recruiting moiety, VH032 was selected, together with pomalidomide and DCAF 15. Differently from CRBN-based PROTACs, VHL-based degraders **114** and **115** (Figure 24) showed weak activity against both BRD7 and BRD9.

**Figure 25:**
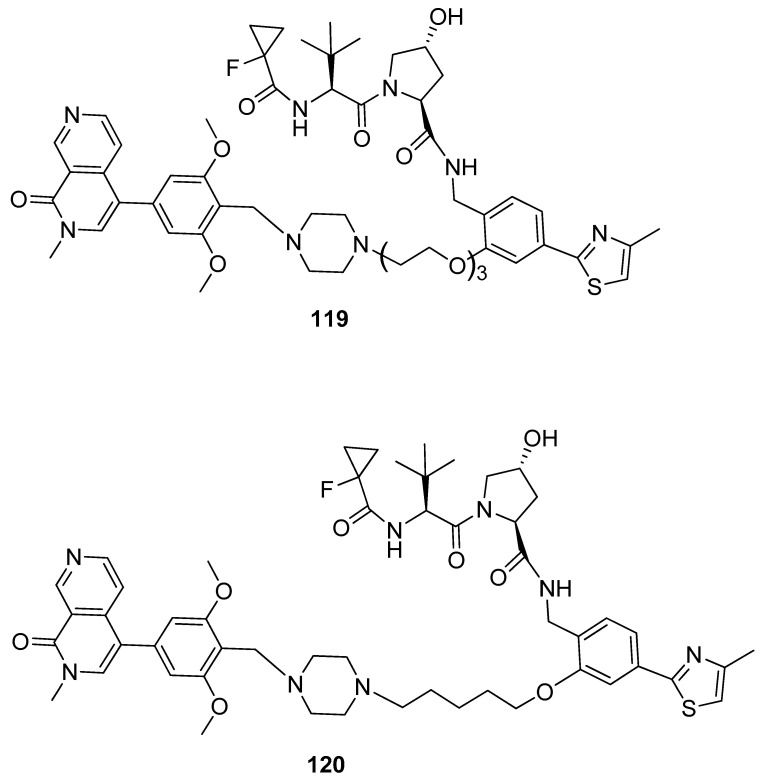
Structures of compounds **119** and **120**.

To enhance the formation of ternary complexes and the degrading activities of **114** and **115**, a second generation of PROTACs was created by altering the linker’s length and composition, as well as the kind of E3 ligase binder used. Positive results were obtained with shorter and more lipophilic linkers. To enhance binding affinity, small modifications to the structure of the VHL ligand were introduced, using alternative warheads (compounds **116**–**118**, Figure 24). The screening for BRD9 and BRD7 degradation after treatment for 4 and 16 h showed compound **119** (Figure 25) as the most active compound, with a half-degrading concentration of 560 nM against BRD9. To further enhance the potency of compound **119**, a new series of compounds was designed, keeping the same warhead of 118 and changing the linker and the BRD7/BRD9 binder. After RI-1 cells were treated with 1 μM of the novel compounds for 2 and 8 h, BRD9 and BRD7 levels were analyzed using Western blot, and 120 was found to be the most potent degrader. Analysis at different concentrations gave a half-maximal degradation concentration (DC_50_) of 1.76 nM and 4.5 nM against BRD9 and BRD7.

**Figure 26:**
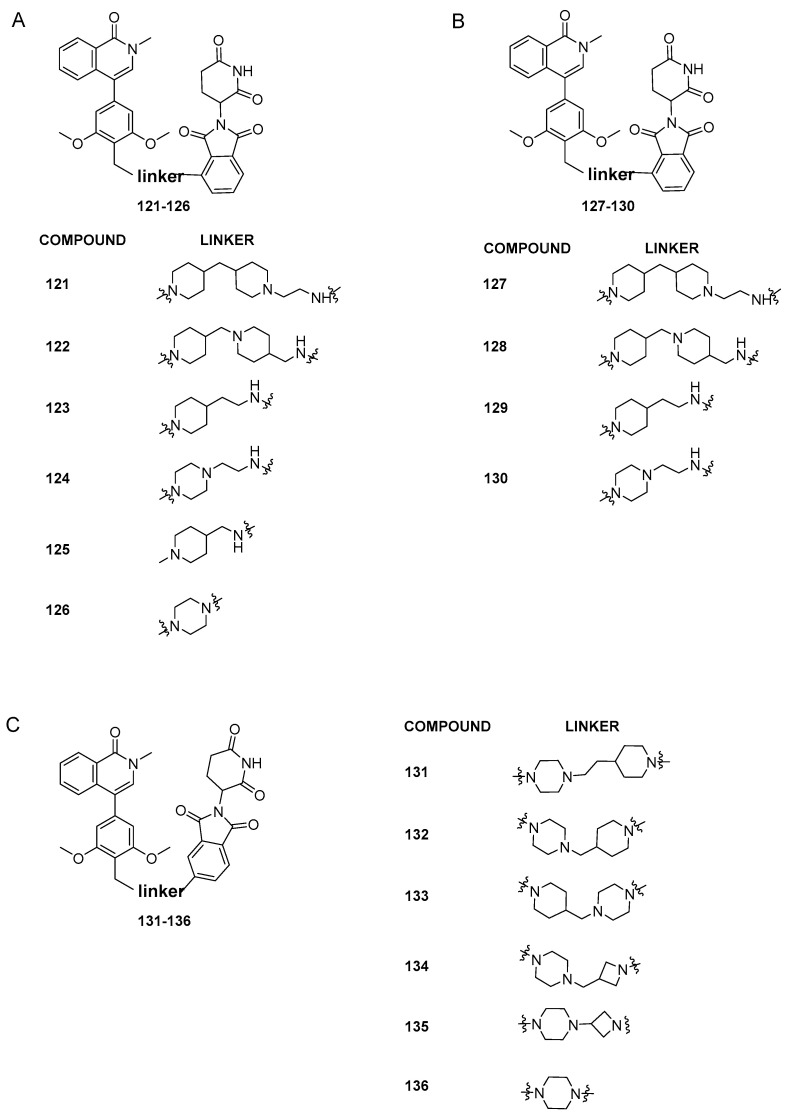
(**A**) Structures of compounds **121**–**126**; (**B**) structures of compounds **127**–**130**; (**C**) structures of compounds **131**–**136**.

Recently, with the aim of obtaining an orally bioavailable BRD9-PROTAC, Zhang et al. [57] reported the synthesis of a new series of BRD9 degraders based on BI-7271 binder (compound **73**) and the cereblon ligand thalidomide. The authors started from the consideration that more rigid linkers increase oral activity, as shown by literature data. They designed the first series of dual binders, with linkers differing in number of rings and polarity, and attached to the C3 of thalidomide (compounds **121**–**126**, Figure 26A). Except for **125** and **126** which displayed poor BRD9 degradation profiles, compounds **121**–**124** gave excellent results in terms of protein degradation activity and cell proliferation inhibition, with IC_50_ values ranging from 3.32 ± 1.91 to 0.06 ± 0.05 nM against MV4-11 cells. The parameters calculated with ADMETlab to predict oral activity indicated compound **123** as the most promising. However, analysis of blood samples after a single oral administration of 20 mg/kg of 123 to ICR mice showed poor oral absorption.

In order to decrease the size of the BRD9 warhead, while retaining the E3 ligand and the best linkers from the first series (Figure 26A), a new series of degraders was created (compounds **127**–**130**, Figure 26B). In comparison to the related compounds **121**–**126**, the degradation activity experiments on **127**–**130** showed a decrease in potency, indicating the significance of the benzene ring structure in the BRD9 binding moiety for preserving pharmacological efficacy. The last series of compounds (Figure 26C) was then designed, keeping the BRD9 binder of the first series and connecting the linkers to the C-4 position of thalidomide. Among all the synthesized compounds (**131**–**136**), **136** demonstrated the most potent proliferation suppression and degradation activity with an IC_50_ of 3.69 ± 3.58 nM in MV4-11 cells and a degradation rate of 93% at 10 nM and 99% at 100 nM. Interestingly, in MV4-11 cells, BRD4 and BRD7 proteins did not degrade at a dose of 100 nM, suggesting that C6 is selective for BRD9 protein. Lastly, **136** oral activity was studied in ICR mice, which showed good oral absorption characteristics with a Cmax value of 3436.95 ng/mL.

**Figure 27:**
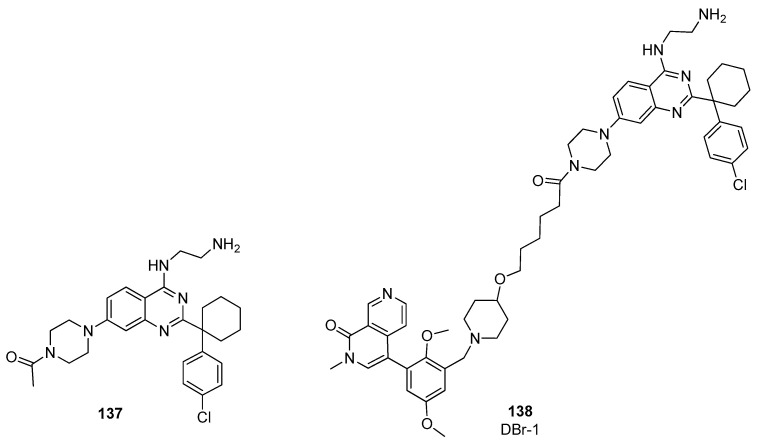
Structures of compounds **137**–**138**.

Recently, DCAF1 (also known as Vpr-binding protein (VprBP)) [58] was identified as a new interesting E3 ligase receptor for targeted protein degradation. Schröder et al. took advantage of a DCAF1 binder **137** previously developed by their group (Figure 27), [59] to design a new BRD9 PROTAC targeting this ligand [60]. The piperazine group attached to the quinazoline core was used to anchor an aliphatic carbon linker. As BRD9 binder, the structure of BI-9564 [39] was used, affording DBr-1 (compound **138**, Figure 27).

DBr-1 showed a slightly lower degrading potency with respect to VZ185 and dBRD9, with a DC_50_ value of 90 nM, and a selectivity for BRD9 over BRD7 similar to dBRD9. These results prove that E3 ligase receptor DCAF1 can be a useful target alternative to CRBN and VHL for the preparation of new PROTACs, with the aim of reducing the possibility of ligase-specific resistance mechanisms.

## 5. Conclusions

BRD9 inhibition can lead to the modulation of gene expression, potentially creating a more controlled and regulated cellular environment. This modulation proves to be beneficial in influencing specific pathways related to disease or cellular processes. Successful BRD9 inhibition could open up new avenues for therapeutic interventions, providing alternative treatment options for conditions associated with dysregulated chromatin remodeling.

BRD9 binders prove to be promising as potential therapeutics for a variety of malignant cancers, above all AML and MRT. Preclinical studies have demonstrated the efficacy of BRD9 inhibition in suppressing tumor growth, enhancing the sensitivity of cancer cells to chemotherapy, and modulating immune responses in inflammatory conditions.

However, some BRD9 inhibitors may lack the desired selectivity, affecting other bromodomain-containing proteins and causing off-target effects. This lack of specificity can limit the therapeutic potential of such inhibitors. Furthermore, despite the role of BRD9 in the related remodeling complex, compounds demonstrating robust activity on isolated BRD9 protein may not necessarily yield favorable results in cell assays. To enhance cellular activity and, consequently, the biological effect, the most promising binders are currently being utilized as a starting point for the development of PROTACs.

Recently, two BRD9 degraders entered clinical trials. FHD-609, developed by Foghorn Therapeutics, Inc., was in a phase 1 trial (NCT04965753) in patients with advanced synovial sarcoma and SMARCB1-deleted tumors. Unfortunately, FDA has placed a partial clinical hold on the phase 1 trial after adverse effects. On the other end, C4 Therapeutics, Inc also discontinued phase 1 clinical trial of CFT8634 (NCT05355753), a potent and selective oral heterobifunctional degrader of BRD9 for the treatment of synovial sarcoma and SMARCB1-deleted tumors, since BRD9 degradation did not produce sufficiently effective results in patients.

In conclusion, despite the prospect of BRD9 binders and degraders as therapeutic agents, several challenges remain to be addressed. Continued research efforts are needed to fully understand the therapeutic implications of targeting BRD9 and to realize the clinical potential of these compounds in treating various diseases.

## Figures and Tables

**Table 1:** Specific substrates for the subfamily IV of BRDs.

Bromodomain	Substrate	Reference
ATAD2	H3K9ac, H3K14ac, H4K5ac, H4K12ac	[8]
BRD1	H3K14ac, H4K5acK8ac, H4K5prK8pr	[6,7,8]
BRD9	H4K5acK8ac, H4K5prK8pr, H4K5buK8bu	[9]
BRD7	H3K9ac H3K14ac H4K8ac H4K12ac H4K16ac	[10]
BRPF1	H2AK5ac H3K14ac H4K8ac H4K5ac H4K12ac	[11]
BRPF3	H3K14ac	[12]
KIAA1240	Not determined	

**Table 2:** FRAP assay results ^(a)^ and cytotoxicity results ^(b)^.

Compound	Cell Line	Conc (µM)	FRAP Recovery Time	Cytotoxicity
**5** (LP99) ^(a)^	U2OS	0.8	0.8	-
**5** (LP99) ^(b)^	U2OS	≤33	-	no
**13** ^(a)^	U2OS	1	0.8	-
**19** ^(a)^	U2OS	1	~5	-
**20** ^(a)^	U2OS	1	~5	-
**21** ^(a)^	U2OS	1	~3	-
**22** (Bromosporine) ^(a)^	U2OS	1	1	-
**31** ^(b)^	HEK293	≤33	-	no
**75** (BI-7273) ^(a)^	U2OS	1	~0.70	-
**79** (BI-9564) ^(a)^	U2OS	0.1	~0.80	-

**Table 3:** Antiproliferative biological activity for the most promising compounds.

Compound	Cell Line	IC_50_ (µM)	EC_50_ (µM)	GI_50_ (µM)
**22** (Bromosporine)	MV4;11	0.5793	n.d	n.d
	KASUMI-1	0.2067	n.d	n.d
	OCI-AML3	0.3990	n.d	n.d
**44**	CRF-CEM	50 ± 5	n.d	n.d
	K-562	90 ± 5	n.d	n.d
	HL-60	>100	n.d	n.d
	Kasumi1	97 ± 4	n.d	n.d
	THP-1	95 ± 6	n.d	n.d
	HaCaT	>100	n.d	n.d
**51**	CCRF-CEM	35 ± 4	n.d	n.d
	K-562	65 ± 4	n.d	n.d
	HL-60	81 ± 5.5	n.d	n.d
	Kasumi1	72 ± 5	n.d	n.d
	THP-1	60 ± 5	n.d	n.d
	HaCaT	>100	n.d	n.d
**61** (I-BRD9)	NB4	n.d	n.d	n.d
	MV4-11	n.d	n.d	n.d
	SU-DHL4	n.d	n.d	n.d
**75** (BI-7273)	EOL-1	n.d	1.4	n.d
**79** (BI-9564)	EOL-1	n.d	0.8	n.d
**84**	EOL-1	1.76 ± 0.05	n.d	n.d
	A549	6.12 ± 0.18	n.d	n.d
**88**	SNB-75	n.d	n.d	0.182
	UO-31	n.d	n.d	0.479
	CAKI-1	n.d	n.d	0.708
	DU-145	n.d	n.d	13.2
	SW-620	n.d	n.d	12.0
	NCI-H460	n.d	n.d	10.2
**96**	Jurkat	110 ± 9	n.d	n.d
	MCF-7	>500	n.d	n.d
	A375	450 ± 16	n.d	n.d
	Caco2	300 ± 14	n.d	n.d
	HaCaT	>500	n.d	n.d
**97**	Jurkat	145 ± 11	n.d	n.d
	MCF-7	>500	n.d	n.d
	A375	270 ± 18	n.d	n.d
	Caco2	300 ± 12	n.d	n.d
	HaCaT	>500	n.d	n.d

## Data Availability

Not applicable.
